# Preparing Physicians for Modern Health Systems: Embedding Leadership and Hospital Administration Training in Graduate Medical Education With Lessons From Psychiatry

**DOI:** 10.7759/cureus.110963

**Published:** 2026-06-16

**Authors:** Bhargav Patel, Abigail Sojka, Rajeev Krishna, Todd Peters, Jeffrey Hunt

**Affiliations:** 1 Department of Psychiatry and Health Behavior, Brown University, Providence, USA; 2 Strategy, Planning, and Execution, Sheppard Pratt Health System, Towson, USA; 3 Medicine, Sheppard Pratt Health System, Towson, USA

**Keywords:** graduate medical education, healthcare systems, hospital administration, physician leadership, psychiatry residency training

## Abstract

Physicians are entering practice in healthcare systems shaped by consolidation, corporatization, administrative complexity, and rising burnout. These pressures are especially relevant in psychiatry and child and adolescent psychiatry, where clinicians must navigate fragmented systems, multidisciplinary teams, reimbursement constraints, and organizational decisions that directly affect patient care. However, most graduate medical education programs provide limited formal training in leadership, hospital administration, finance, governance, or systems-level decision-making. This editorial argues that leadership development and hospital administration exposure should be integrated into residency and fellowship training as core educational experiences. Drawing on a trainee’s psychiatry hospital administration elective at a large nonprofit psychiatric health system, it illustrates how exposure to executive leadership meetings, quality and safety committees, operational dashboards, strategic planning, service line decisions, and electronic health record implementation can help trainees understand how health systems function and where physicians can meaningfully intervene. For psychiatrists and child psychiatrists, leadership training is not only preparation for formal administrative roles. It is central to effective clinical practice, team coordination, patient advocacy, and professional sustainability. A tiered model that provides foundational leadership exposure for all trainees, with advanced elective pathways for interested residents and fellows, may help prepare physicians to engage constructively with modern health systems. Embedding leadership curricula and hospital administration electives into graduate medical education can strengthen physician agency and prepare the next generation to shape, rather than simply endure, the systems in which they practice.

## Editorial

Physicians are entering practice in an environment defined by consolidation, corporatization, and accelerating complexity in healthcare delivery. Consolidation refers to the movement away from independent or small-group practice toward employment within large hospitals, health systems, managed care organizations, and corporate practice models. Corporatization reflects the growing influence of organizational, financial, and non-clinical administrative priorities. At the same time, healthcare delivery has become increasingly complex, requiring physicians to navigate evolving payment models, regulatory requirements, electronic health records, quality metrics, staffing constraints, interdisciplinary teams, and fragmented systems of care. Together, these forces have reshaped the day-to-day experience of clinical practice and increased the need for physicians to understand not only patient care but also the systems in which that care is delivered. High rates of burnout and moral distress also raise concerns about the sustainability of medical careers. Large national surveys have shown that more than half of US physicians report at least one manifestation of burnout, with 53.6% reporting emotional exhaustion at least several times a month or more, compared to 39.1% in the general working population. Daily emotional exhaustion was reported by 12.0% of physicians compared to 5.7% of the general working population. [1]. Another burnout symptom, depersonalization, was reported at once a month or more in 25.7% of physicians compared to 17.9% of the general working population [1]. Using high emotional exhaustion and/or depersonalization scores as indicators of overall burnout, 39.5% of physicians met the criteria for being “burned out” compared to 24.6% of the general working population [1].

These realities sharpen the questions posed by many educators and professional bodies: What can be done to improve the professional experience for physicians, both now and for the next generation? How can the profession maintain a thriving workforce capable of meeting the challenges of today and tomorrow? And how should clinicians and trainees respond to the juggernaut of corporatization that increasingly shapes the clinical environment? These pressures are equally salient in psychiatry and child and adolescent psychiatry, where practice patterns have shifted markedly over the past two decades. Psychiatrists are increasingly employed by large health systems, group practices, or managed care organizations, while fewer trainees pursue solo private practice immediately after training [2].

One underused but promising strategy is explicit physician leadership education, including structured hospital administration electives during residency and fellowship. Such experiences can help trainees understand how systems function, how decisions are made, and how physicians can shape those systems rather than simply endure them.

This editorial draws on a psychiatry trainee’s hospital administration elective at a large nonprofit psychiatric health system and situates that experience within the growing literature on physician leadership. This experience represents a single trainee’s perspective within a well-resourced institution, and its observations may not be directly generalizable to all residency and fellowship programs. Nevertheless, it offers a useful illustrative case for considering how structured exposure to hospital leadership, administration, and systems-level decision-making may help prepare physicians for contemporary practice. We argue that hospital leadership electives and leadership curricula should be considered important strategies for improving the professional experience, preparing physicians for modern healthcare challenges, and equipping the workforce to engage constructively with increasingly complex and corporatized health systems.

Leadership expectations and the training gap

Physicians are increasingly expected to lead teams, steward resources, and participate in organizational decision-making, yet most graduate medical education (GME) programs offer limited formal preparation for these roles. Blumenthal and colleagues described this as a “leadership gap in medicine,” emphasizing that physicians frequently occupy leadership positions without systematic development in leadership skills [3].

A systematic review by Sadowski et al. identified 52 leadership curricula in GME and found wide variation in content, methods, and evaluation. The authors concluded that leadership training is feasible and generally well-received, but is often short, heterogeneous, and insufficiently evaluated at higher educational outcomes [4]. Kumar et al. similarly reviewed leadership training programs for residents and fellows, highlighting that although interest in leadership development is growing, curricula remain fragmented and frequently optional rather than embedded in core training [5].

Psychiatrists and child psychiatrists frequently coordinate multidisciplinary teams that include psychologists, social workers, educators, case managers, and community agencies and often serve as clinical anchors within fragmented systems of care [6]. Viewing leadership training as relevant to all trainees, rather than only those pursuing administrative careers, reflects the reality that effective psychiatric practice already demands leadership competencies, such as communication, systems navigation, and team coordination. Integrating leadership development universally during training may therefore enhance both clinical effectiveness and professional sustainability within psychiatry.

Blumenthal and colleagues further emphasized that residents need structured, longitudinal leadership development, including training in self-awareness, team dynamics, quality improvement, and systems thinking, if they are to meet the demands of modern healthcare [3]. Collectively, these studies point to a mismatch between the leadership demands placed on new attending physicians and the limited exposure they receive during training.

Variability in institutional resources and faculty expertise may limit the feasibility of comprehensive leadership training at smaller or resource-constrained programs. In this context, a standardized national leadership curriculum, developed through professional societies or accrediting bodies, could provide a foundational framework that ensures baseline leadership competencies for all trainees, regardless of program size. Such a curriculum could be supplemented by institution-specific experiential learning, including hospital administration electives or committee participation. Together, a hybrid model that combines national curricular standards with local experiential opportunities and faculty development may offer the most scalable and equitable approach to preparing residents for leadership roles in contemporary healthcare systems.

The rise of physician executives and the value of clinical leadership

Although leadership and management are often used interchangeably, they represent distinct and complementary domains [7]. Leadership involves setting direction, inspiring people, and driving change, shaped by personal principles, values, and relational skills. Management, by contrast, centers on planning, organizing, and implementing organizational vision through processes, systems, structures, and governance. Effective physician executives must develop fluency in both: leadership capacity enables physicians to articulate vision and mobilize teams toward better patient care, while management competency equips them to operationalize that vision through the structures and systems that govern healthcare delivery. Understanding this distinction clarifies the scope of physician training needs. Leadership development that addresses only the inspirational dimensions of the role, without also building management literacy, leaves clinicians underprepared to exercise authority in the operational contexts where health systems function. The hospital administration elective described below was designed with precisely this dual aim: equipping trainees to both lead and manage within complex organizational environments.

In parallel, health systems worldwide have increasingly recognized the value of physician leaders in formal executive roles. Goodall examined top-ranked US hospitals and found a strong positive association between hospital quality rankings and whether the chief executive officer was a physician, suggesting that physician-led hospitals may perform better on certain quality metrics [8]. These perspectives converge on a common theme: physician leadership matters, and the demand for physician leaders is increasing.

Yet many physicians reach mid-career feeling unprepared for the administrative and strategic aspects of leadership roles. Providing leadership education and system-level exposure during residency is a logical response to this gap, particularly if the profession wishes to counterbalance purely corporate or non-clinician control of healthcare organizations.

A psychiatry hospital administration elective: structure and lessons

In response to these needs, one well-resourced New England child and adolescent psychiatry training program developed a hospital administration elective at a large nonprofit psychiatric health system. During this rotation, a child psychiatry fellow, one of the authors of this editorial, stepped away from routine clinical duties to spend dedicated time with institutional leaders, including the chief executive officer, chief operating officer, chief medical officer, chief medical information officer, and service line leaders.

The goals of the rotation were to help the trainee understand and navigate the major disciplines that comprise hospital leadership and administration, develop familiarity with how proposals are evaluated and decisions are made at the C-suite level, and appreciate the role of clinical leaders in bridging patient care with hospital administration.

The rotation included regular participation in executive leadership meetings and strategic planning sessions, attendance at quality and safety committees reviewing patient outcomes, safety events, and regulatory issues, and exposure to scheduling, staffing models, policy discussions, and decisions about opening or closing services. The trainee also reviewed operational dashboards tracking access, length of stay, readmissions, occupancy, and patient experience; participated in discussions with executives and leaders across the organization; and attended meetings with the electronic medical records vendor alongside administrative, clinical, and information technology leadership. Along with the rotation, the trainee was able to maintain responsibilities with the home organization through a combination of virtual visits with longitudinal therapy and clinic patients and blocked time for continued research responsibilities.

From the trainee’s perspective, several themes emerged from the elective. First, the experience helped reveal the system behind every clinical encounter. System problems that often become personal frustrations for clinicians, such as a lack of inpatient psychiatric beds, long emergency department boarding, or difficulty coordinating with community agencies, were reframed as issues involving capacity management, financing, staffing, contracting, and regional policy. Observing leaders interpret trends and constraints helped connect frontline clinical experience to the structural determinants of care. This broader view may reduce feelings of helplessness and moral injury by showing that many persistent problems have identifiable upstream causes and potential levers for change.

Second, the elective clarified how organizational decisions are made and where physicians can meaningfully intervene. Executive meetings revealed how leaders balance patient safety, access, financial viability, regulatory requirements, and staff well-being. Strategic choices about service lines, technology investments, and workforce models were made through iterative review of data and stakeholder input. Seeing this process demystified administration and highlighted where clinician perspectives are most needed, such as anticipating the clinical consequences of throughput decisions or designing workflows that preserve therapeutic relationships.

Third, immersion in leadership spaces, combined with explicit mentorship from physician and non-physician leaders, helped the trainee begin to see leadership as part of professional identity rather than as a distant, title-based role. This shift is particularly important in psychiatry, where clinicians frequently lead multidisciplinary teams, navigate complex interfaces with social services and schools, and advocate for under-resourced populations. Early leadership identity formation may make such responsibilities more sustainable and meaningful.

Educators and program planners can support this goal by embedding leadership competencies into milestones and evaluations so that they are recognized as core professional skills. Although relevant concepts currently exist in psychiatry milestones, particularly within systems-based practice subcompetencies related to system navigation for patient care and the physician’s role in health care systems, many trainees may still feel inadequately prepared by current residency and fellowship training methods. Programs can further strengthen this preparation by developing structured hospital administration electives with clear objectives, experiential components, and mentored projects, while aligning curricula with existing evidence on effective leadership training, including longitudinal formats and project-based learning.

Multiple systematic reviews have shown that leadership curricula can improve self-assessed leadership skills, systems thinking, and understanding of healthcare operations during GME [4,5]. However, this is still vastly underutilized in practice during residency and fellowship training. Barriers include limited protected time within already dense clinical schedules, a shortage of faculty with formal leadership training, competing educational priorities, and the absence of standardized, evidence-based leadership curricula. As a result, leadership education is often treated as optional or extracurricular rather than as a core component of physician training.

Addressing corporatization through physician leadership

The juggernaut of corporatization reflects structural shifts toward large health systems, private equity ownership, and non-clinician control of healthcare organizations. These shifts can create misalignment between financial imperatives and professional values, contributing to burnout and feelings of lost autonomy. Leadership training alone cannot reverse these trends, but it can change how physicians engage with them.

The growth of private equity-backed behavioral health organizations and large health system employment models has shifted many psychiatrists from autonomous practice into settings where clinical policies, staffing ratios, and care models are determined by non-clinician administrators [2]. These dynamics heighten the need for psychiatrists and child psychiatrists to possess leadership and systems literacy skills that enable informed advocacy for patient-centered care within increasingly corporatized organizational structures.

Beyond the dynamics of corporatization, psychiatry faces a distinctly challenging financial landscape that compounds these pressures. Psychiatric visits are reimbursed at substantially lower rates than those in most other medical specialties, reflecting longstanding parity gaps that persist despite legislative efforts to address them [9]. The prevailing fee-for-service reimbursement model further disadvantages care delivery models such as collaborative care and coordinated care, which distribute clinical effort across teams and settings in ways that are not easily captured in traditional billing structures. Although value-based payment models are gradually reshaping reimbursement across medicine, behavioral health has lagged behind this transition. These structural financial constraints mean that psychiatrists frequently operate in resource-constrained environments where clinical decisions are shaped as much by reimbursement logic as by clinical evidence [9]. For physician leaders in psychiatry, understanding the financial architecture of care delivery is therefore not merely a business competency but a prerequisite for effective advocacy, resource stewardship, and sustainable practice within systems increasingly defined by economic pressures.

When physicians are literate in finance, governance, and health policy, they are better positioned to negotiate and advocate around productivity metrics, staffing, and service line design. Cultivating physician leadership is not a luxury, but a necessity if physicians are to shape, rather than simply endure, corporatized systems.

An important related question is whether leadership training should be targeted to a subset of trainees or broadly applied across GME. While not all physicians will pursue formal leadership roles, all trainees benefit from foundational exposure to leadership concepts such as systems thinking, team dynamics, and organizational decision-making. Universal exposure during residency and fellowship can normalize leadership as a core professional competency and equip all physicians to function effectively within complex systems. At the same time, more intensive leadership development may be appropriate for trainees who demonstrate early interest or aptitude, such as those drawn to quality improvement, education, advocacy, or administrative roles. Rather than relying on early self-selection or subjective identification of “leadership potential,” a tiered approach combining broad foundational training for all trainees with optional advanced pathways may be the most equitable and effective strategy. This model allows leadership capacity to emerge organically over time while ensuring that no physician enters practice without the basic skills needed to engage with organizational and corporate structures that shape patient care.

Hospital administration electives during residency and fellowship, or embedded leadership curriculum, can serve as early incubators for such leaders and signal that physicians are expected, and prepared, to participate in organizational decision-making. Clinicians and current trainees can advocate for administrative electives within their programs, seek mentorship from physician leaders, and participate in committees on quality, safety, equity, and electronic health record optimization.

Educators and program planners can embed leadership competencies into milestones and evaluations, ensuring they are recognized as core professional skills. Related competencies already appear in the ACGME Psychiatry Milestones, particularly within Systems-Based Practice 2, “System Navigation for Patient-Centered Care,” and Systems-Based Practice 3, “Physician Role in Health Care Systems” [10]. However, the presence of these competencies within assessment frameworks does not necessarily ensure structured exposure to hospital administration, finance, governance, or executive-level decision-making during residency and fellowship. Programs can further strengthen this preparation by developing structured hospital administration electives with clear objectives, experiential components, and mentored projects, while aligning curricula with existing evidence on effective leadership training, including longitudinal formats and project-based learning [4,5]. However, leadership curricula and hospital administration electives also present important implementation challenges. Residency and fellowship schedules are already dense, and adding leadership content without protected time risks increasing trainee burden or displacing essential clinical experiences. Programs may also lack faculty with formal administrative or leadership training, particularly in smaller or resource-constrained institutions. In addition, leadership curricula can become overly theoretical if not connected to real organizational problems, or overly idiosyncratic if dependent on a single mentor or local institutional culture. These challenges can be mitigated by using a tiered approach that provides foundational leadership content to all trainees while offering more intensive electives for interested residents and fellows. Programs can also incorporate protected elective time, clear learning objectives, mentored projects, faculty development, standardized curricular resources, and partnerships with health system leaders. Aligning leadership training with existing systems-based practice milestones may further reduce curricular redundancy and help ensure that leadership development complements, rather than competes with, core clinical training.

Health system leaders can partner with training programs to open executive spaces to residents and fellows, co-create leadership tracks, and support trainee participation in system-level initiatives that address access, quality, and clinician well-being. Future research should evaluate the effectiveness of leadership and hospital administration electives using outcomes that extend beyond trainee satisfaction. Studies could examine whether these experiences improve leadership self-efficacy, systems-based practice competencies, understanding of hospital operations, engagement in quality improvement, and readiness for clinical-administrative roles. Longitudinal research may also clarify whether early exposure to health system leadership influences career trajectories, physician agency, burnout, retention, and participation in institutional decision-making. In psychiatry specifically, future work should explore how leadership training affects interdisciplinary collaboration, advocacy within fragmented systems of care, and the ability of physicians to shape care models in increasingly complex and corporatized practice environments.

Conclusion

The professional experience of physicians is shaped not only by individual resilience but by the systems in which they work. To maintain a thriving physician workforce and confront the challenges of corporatization as well as practice in private, community, and government spaces, the profession needs physicians who are prepared to lead.

Hospital administration electives, like the leadership elective described here, offer a concrete way to introduce trainees to executive-level thinking and decision-making. Coupled with structured leadership curricula and faculty development, such experiences can improve professional agency, foster realistic leadership identities, and prepare tomorrow’s psychiatrists and other physicians to shape the systems in which they and their patients must live and work.
